# The New Pharmacopeia

**Published:** 1905-02

**Authors:** 


### THE NEW PHARMACOPEIA.

There met in Brussels in the summer of 1902 an important congress of physicians and pharmacists. America was represented by Dr. Horatio C. Wood and Dr. Frederick P. Power, each delegate from each country being appointed by the respective governments.

*By Dr. J. H. Stucky, (Journal American Medical Association, October 15, 1904).

The work of the congress was important, the idea being to frame an international pharmacopeia of patent remedies. The development of this congress was slow, the suggestion assuming shape in 1893 at the Seventh International Pharmaceutical Congress, which met that year in Chicago, during the period of the World's Fair. It was a wise move to exclude all medicinal substances, for each nation has its individual drugs, and it would be difficult to displace or supplant these.

The United States Pharmacopeia will be the first national authority to recognize the standards set by this congress. Professor Remington, the able chairman of the Committee of Revision, read a report on this subject before the section on pharmacology at the recent meeting of the American Medical Association; this report was published in the *Journal of the American Medical Association*, published December 3, 1904. This eminent authority states that he has received many letters of inquiry on the subject, and his paper is a resumé of the subject.

The present state of pharmacopeia is confusing to say the least, and the Committee of Revision has had its trials and problems. Especially has this been true in regard to the newer synthetics. No sooner does a physician begin to use one new drug than he is importuned to discard it and try a later one. The procession is interminable and grows in size as it passes the reviewing stand.

The pharmacopeia 1890 was found to be too rigorous, and hypercritical in actual practice. There is no necessity for ordering chemical salts to be microscopically or analytically free from harmless substances, if such amounts are known and allowed for; on the part of the manufacturer it grew to be a serious problem, for it increased the cost of production often over one hundred per cent. Chemical tests are now very precise, and can be used with entire safety to patient and practitioner. So the committee has established the principle that it is better to introduce standards which can be easily attained, and then look to the law to prosecute offenders. Thus boric acid must be 99.8 per cent. pure, which does not prohibit the manufacturer from making it of one hundred per cent. purity, but does prevent boric acid of ninety per cent. grade being used in medicine. Again a clearcut distinction will be made between drugs used commercially and medicinally. The present standard bears hard on the manufacturer of drugs used in the arts and commerce.

Taking arsenic as a type the new rules require that every official arsenical solution throughout the world shall have the strength of one per cent. Ten per cent. is the standard for tincture of opium, five per cent. of ferrous iodide in the syrup of the iodide of iron. Ten and twenty per cent. tinctures will be required, ten per cent. for patent drugs, twenty for weaker. This will cut down the strength of many tinctures, such as the tincture of aconite.

The alteration in nomenclature will not be great; but serious errors will be corrected, such as the term carbolic acid. This drug is not an acid at all, but a phenol, "carbolic acid" will be used as a synonym for the present, but the up-to-date title will be "phenol." The committee, however, advise the rejection of all synonyms in prescription writing, such as "cold cream," "laudanum," "paregoric," "Goulard's cerate," etc.

In the synthetics the definite chemical name will be used, rather than the titles given by the manufacturers. This will be a little hard at first, but the physician must rise to the improvement. Again for the first time the average dose is given, not the maximum one, however, as this varies greatly under different circumstances.

Three months will be allowed after the publication of the pharmacopeia before these improvements will go into effect in order to allow physicians, druggists and manufacturers to prepare for them.

